# Three-month treatment outcome of medication-overuse headache according to classes of overused medications, use of acute medications, and preventive treatments

**DOI:** 10.1038/s41598-024-66906-0

**Published:** 2024-07-11

**Authors:** Sun-Young Oh, Jin-Ju Kang, Hong-Kyun Park, Soo-Jin Cho, Yooha Hong, Mi-Kyoung Kang, Heui-Soo Moon, Mi Ji Lee, Tae-Jin Song, Young Ju Suh, Min Kyung Chu

**Affiliations:** 1 Department of Neurology, Jeonbuk National University Hospital & School of Medicine, Jeonju, South Korea; 2 Research Institute of Clinical Medicine of Jeonbuk National University-Jeonbuk National University Hospital, Jeonju, South Korea; 3 Department of Neurology, Inje University Ilsan Paik Hospital, Inje University College of Medicine, Seoul, South Korea; 4 Department of Neurology, Dongtan Sacred Heart Hospital, Hallym University College of Medicine, Hwaseong, South Korea; 5 Department of Neurology, Kangbuk Samsung Hospital, Sungkyunkwan University School of Medicine, Seoul, South Korea; 6 Department of Neurology, Seoul National University Hospital, Seoul National University College of Medicine, Seoul, South Korea; 7 Department of Neurology, Seoul Hospital, Ewha Womans University College of Medicine, Seoul, South Korea; 8 Department of Biomedical Sciences, College of Medicine, Inha University, Incheon, Korea; 9 Department of Neurology, Severance Hospital, Yonsei University College of Medicine, Seoul, 03722 South Korea

**Keywords:** Medication-overuse headache, Migraine, Chronic headache, Treatment strategies, Three-month follow-up, Diseases, Health care, Medical research, Neurology

## Abstract

Medication overuse headache (MOH) is a chronic headache disorder that results from excessive use of acutely symptomatic headache medications, leading to more frequent and severe headaches. This study aims to assess the 3-month treatment outcomes in MOH patients, focusing on the types and usage of overused medications, as well as preventive treatments. This prospective cross-sectional study analyzed the treatment outcomes of 309 MOH patients from April 2020 to March 2022. Patients were advised to discontinue overused medications immediately and offered preventive treatments based on clinical judgment. Data on headache characteristics, medication use, and impact on daily life were collected at baseline and 3 months. Results showed overall significant improvements in headache-related variables in patients completing the 3-month treatment follow-up. The median number of headache days per month decreased from 15 days at baseline to 8 days after 3 months (*p* < 0.001). Patients who overused multiple drug classes demonstrated increased disability levels (mean Headache Impact Test-6 score: 62 at baseline vs. 56 at 3 months, *p* < 0.01). Those who continued overusing medications reported more days of severe headache (mean 18 days at baseline vs. 14 days at 3 months, *p* < 0.05) and greater impact (mean Migraine Disability Assessment score: 35 at baseline vs. 28 after 3 months, *p* < 0.05) compared to the baseline. Differences in headache outcomes were evident across different preventive treatment groups, with generalized estimating equation analyses highlighting significant associations between clinical characteristics, overused medication classes, and preventive treatments. Most MOH clinical features significantly improved after 3 months of treatment. However, notable interactions were observed with certain clinical presentations, suggesting possible influences of overused medication classes, usage patterns, and preventive treatment types on MOH treatment outcomes. This study underscores the importance of individualized treatment strategies and the potential benefits of discontinuing overused medications.

## Introduction

Medication-overuse headache (MOH), a chronic headache disorder arising from the prolonged use of acute symptomatic headache medications, resulted in the development of headaches that are more frequent, severe, and difficult to treat^1^. Because headaches secondary to medication overuse were first reported in 1951 in the context of ergotamine^2^, excessive use of the therapeutic agents has been recognized to exacerbate pre-existing headaches and promote headache chronification^3^. MOH was first introduced in the International Classification of Headache Disorders, 1st Edition (ICHD-1) in 1988 as “drug-induced headache” and was later renamed “MOH” in ICHD-2 in 2004 and retained in ICHD-3 in 2018^4–6^.

The importance of effective treatment strategies for MOH is becoming more widely recognized in the neurology and headache communities^3^. Therefore, establishing effective treatment strategies to reduce the patients’ burden and alleviate the socioeconomic impact of MOH is of great importance. Despite the large controversies regarding the detailed strategies of MOH treatment, most guidelines recommend complete discontinuation of overused medications, switching to medications with low MOH risk, and patient education^7–9^. Prognostic factors of MOH treatment reportedly include the severity of primary headaches, duration of overuse of medications, and previous failure of preventive treatment^10–12^. Nevertheless, data on the causal relationship between drugs of overuse and clinical characteristics following MOH treatment are limited, and the relationship between the use of overused drugs and clinical characteristics following preventive treatment is scarce^13^.

Recently, as part of the Registry for Load and Management of MEdicAtion OveruSE Headache (RELEASE) study, we reported that some clinical characteristics of MOH differed significantly according to the classes of overused medications at baseline^14^. Patients who overused multiple drug classes presented with more severe clinical characteristics, including a shorter interval between chronic daily headache (CDH) and MOH onset, more days of using acute medications, and more emergency room visits compared with the MOH groups who overused single medication classes^14^. From the results, we can infer that the clinical progression of MOH may vary depending on the classes of overused medications and the treatment approach. In particular, the frequency of MOH patients overusing multiple classes of drugs (30.1%) and triptans (21.8%) is high, making the class of overused medication an important issue in MOH management^14^. Furthermore, follow-up studies have not previously reported differences in clinical characteristics based on the usage of overused medications and preventive treatments. Therefore, in the current study, we aimed to assess the 3-month treatment outcomes of MOH patients according to the classes and usage of overused medications, as well as types of preventive treatments, utilizing follow-up data from the RELEASE study. We hypothesized that significant variations in the 3-month outcomes would be observed based on the classes of overused medications, the pattern of their usage, and the type of preventive treatment administered.

## Methods

### Study design and participants

We performed a follow-up analysis as part of the RELEASE study, a longitudinal, prospective, cross-sectional observational study of MOH patients in seven neurology clinics in the Republic of Korea from April 2020 to March 2022. Patients were assessed using structured questionnaires and case report forms, and followed 1, 3, 6, and 12 months after the first visit. Patients who met the eligibility criteria were informed about the study during their initial visit, and if they expressed their voluntary willingness to participate, they were enrolled in the study. In our clinical settings, patients can visit primary care physicians or pharmacies at any time.

The protocol for RELEASE has been previously described^15^, and the inclusion criteria for the current study were as follows: (i) age ≥ 18 years, (ii) fulfillment of the MOH criteria in the ICHD-3^16^, (iii) ability to communicate and complete questionnaires, and (iv) provision of written informed consent. All primary and secondary headache disorders associated with MOH were considered for enrollment. MOH was subdivided according to the classes of overused medications: MOH from overuse of ergotamine (code 8.2.1, E-MOH), triptans (code 8.2.2, T-MOH), non-steroidal anti-inflammatory drugs (code 8.2.3, N-MOH), opioids (code 8.2.4, O-MOH), combination analgesics (code 8.2.5, C-MOH), and multiple drug classes that were not individually overused (code 8.2.6, M-MOH). Patients who overused more than one medication class were classified as having M-MOH. In a real-world observational study tracking MOH treatment outcomes, patients who were prescribed barbiturates, benzodiazepines, or opiates, which are rarely used for headache treatment in Korea, were excluded. None of the patients with C-MOH had overdosed on barbiturates medications, and none had O-MOH. Additionally, individuals with unclear identification of the types and dosages of acute medications were not recruited.

The exclusion criteria were as follows: (i) current treatment of medical, neurological (excluding headaches), or psychiatric illness, (ii) inability to provide headache history and drug profile information, (iii) inability to cooperate in filling out questionnaires, and (iv) disagree to undergo follow-up. At the baseline and 3-month visits, the participants were assessed using a standardized case report form and structured self-administered questionnaire. All participating MOH patients were advised to immediately and completely discontinue overused medications, take preventive medications as needed (based on clinician judgment), and use alternative rescue medications. In addition to oral preventive medications, anti-calcitonin gene-related peptide (CGRP) monoclonal antibody (mAb) and onabotulinumtoxinA (OBT-A) were used at the treating physicians’ discretion. The data collected for analysis at baseline and 3-month follow-up included demographic data, classes of overused medications, and headache-related features including headache and severe headache days per month, headache intensity, disability, and the impact of headache. Crystal-clear days per month were defined as the number of days without headaches per month. For healthcare use, we examined the number of hospital and pharmacy visits per month and the number of days using acute medications per month. It was standardized that each month consisted of 30 days.

The Headache Impact Test-6 (HIT-6), Migraine Disability Assessment (MIDAS), and Migraine-Specific Quality of Life Scale (MSQ) were used to gauge the impact and disability caused by headaches and their effects on the quality of life^14,17,18^. Depression and anxiety were examined using the Patient Health Questionnaire-9 (PHQ-9) and Generalized Anxiety Disorder-7 (GAD-7)^19,20^. We examined these outcomes of MOH at baseline and 3 months post-treatment to investigate the impact of classes and usage of overused medications, as well as preventive treatments, over a 3-month period.

### Use of overused medications

Patients were instructed to immediately discontinue the use of overused acute medications from the time of enrollment to ensure a complete cessation of acute medication overuse. Patients who successfully discontinued the overuse of acute medications were defined as the discontinuation group. The reduction group consisted of patients whose monthly frequency of acute medication intake decreased over the following 3 months compared with that at baseline. Patients who remained on overused acute medications without reduction were classified into the maintenance group.

### Types of preventive treatment

All patients were informed of the availability of preventive treatments, and the selection of preventive treatments was based on patient preferences and the decisions of the participating investigators. Preventive treatments in this study were categorized as follows: OBT-A, anti- CGRP mAb, combination of OBT-A and anti-CGRP mAb, oral preventive medication only, and no-treatment groups. The oral preventive medications included a range of options such as antiepileptic drugs, beta-blockers, calcium channel blockers, tricyclic antidepressants, serotonin-norepinephrine reuptake inhibitors, and angiotensin receptor blockers. These were prescribed to all patients except those who opted out of preventive treatment. Adjustments to oral preventive treatments were made if patients experienced intolerable side effects or requested changes.

### Ethic approval and patient consent

This study was reviewed and approved by the Institutional Review Board of each participating center, including the Jeonbuk National University Hospital (IRB 2020-06-028-003). The study was conducted in accordance with the Strengthening the Reporting of Observational Studies in Epidemiology (STROBE) guidelines^21^. The patients provided written informed consent prior to participation. All methods were carried out in accordance with relevant guidelines and regulations including the Declaration of Helsinki and its following amendments.

### Statistical analysis

This descriptive prospective observational study included patients with MOH referred to headache centers in South Korea as a part of the RELEASE study. This study was a secondary analysis of the RELEASE data. The RELEASE study aimed to enroll a minimum of 341 patients, based on a rationale described previously. As an observational study, this analysis did not have a predefined sample size based on statistical power calculations. The hypothesis testing was conducted using a two-tailed approach to assess potential differences in the 3-month treatment outcomes of MOH patients based on the classes and usage of overused medications and types of preventive treatments. This approach allows for the consideration of both increases and decreases in treatment outcomes associated with varying factors. The Kolmogorov–Smirnov test was used to assess variable distribution normality. For non-normally distributed variables, we used the Wilcoxon signed-rank test to compare the baseline and 3-month follow-up data for each overuse group. Pearson’s chi-square test was performed for categorical variables. For our non-normally distributed longitudinal data, we adopted the Generalized Estimating Equation (GEE) method to evaluate the influence of variables such as classes and use of overused medications and preventive treatments, adjusting for patient baseline variables including age, sex, anxiety, and depression^22^. We also calculated means, standard deviations, and 95% confidence intervals for outcomes using GEE with post-hoc analysis. Differences in clinical characteristics according to the classes and use of overused medications and preventive treatments after the 3-month treatment were analyzed using GEE with post hoc analysis. GEE statistical analysis is useful when dealing with related or repeated measurements in data^23^. It’s handy for studies with multiple observations for the same individuals or when considering correlations between groups^24^. GEE takes into account the correlation structure of the data, making it robust to outliers and non-normal distributions. It's also effective for handling clustered or panel data^23,24^. GEE tends to provide more accurate and efficient results compared to traditional linear models^24^. Multicollinearity was assessed using the variance inflation factor (VIF), which was significant with VIF > 10 and no multicollinearity. Non-repeated measures involving non-normally distributed variables were compared using the Kruskal–Wallis test, followed by post hoc pairwise comparisons with a Bonferroni-corrected Mann–Whitney U test. Data are presented as a median with an interquartile range, with a two-tailed *p*-value < 0.05 considered statistically significant. All analyses were performed using SPSS software version 22 (IBM, Armonk, NY, USA).

## Results

### Participants and baseline clinical characteristics

**Figure 1:**
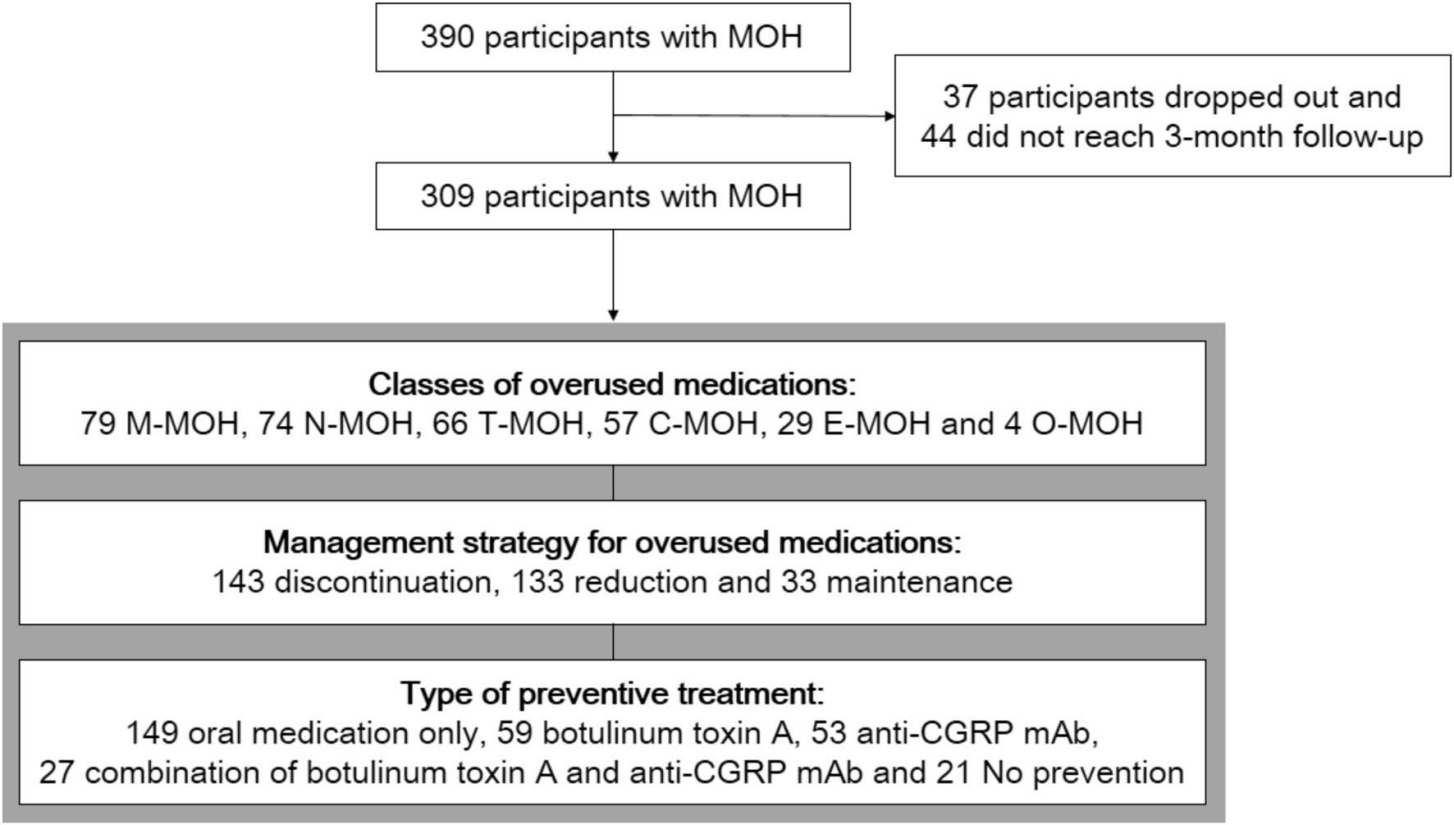
Flow diagram for study participants. *C-MOH* combination analgesic-overuse MOH, *E-MOH* ergotamine-overuse MOH, *M-MOH* MOH attributed to multiple drug classes, *N-MOH* non-steroidal anti-inflammatory drug-overuse MOH, *MOH* medication-overuse headache, *T-MOH* triptan-overuse MOH, *OBT-A* onabotulinumtoxinA, *Anti-CGRP mAb* anti-CGRP monoclonal antibody.

The RELEASE study enrolled 390 MOH patients between April 2020 and March 2022. Of them, 37 (9.5%) dropped out before the 3-month follow-up, and 44 (11.2%) did not reach this milestone (Fig. 1). Thus, 309 participants’ data from the 3-month follow-up were included in our analysis; no missing data were observed. The median age of participants was 45 years (range, 35.5–57.0 in the 25–75th percentiles), and 85.1% (263 participants) of them were female. The most common associated headache type in MOH patients was migraine (97.5%, 301/309), followed by tension-type headache (1.9%, 6/309) and unclassified primary headache disorder (0.6%, 2/309).

Table 1 shows a comparative analysis of baseline variables in MOH patients by classes and use of overused medications, and preventive treatment. The sex distribution was consistent across the five groups of overused medications, three groups of use of overused medications, and five preventive treatment groups. However, the age distribution varied according to the medication class. Specifically, the T-MOH (43.0 [33.5–52.5] years) and N-MOH groups (39.5 [34.5–52.0] years) were significantly younger than the C-MOH (48.0 [36.5–62.5] years, *p* = 0.046, *p* = 0.025) and M-MOH groups (48.0 [38.0–58.0] years, *p* = 0.024, *p* = 0.010). The T-MOH group had fewer monthly headache days, severe headache days, days with acute medications, and pharmacy visits, along with more crystal-clear days at baseline. When evaluated according to the types of preventive treatment, the no-preventive treatment group had more crystal-clear days and fewer headache days those using preventive treatments. Likewise, the oral medication-only and no-treatment groups exhibited lower MIDAS scores but higher MSQ scores and headache-related clinical measures than the OBT-A and anti-CGRP mAb groups. In contrast, the OBT-A, anti-CGRP mAb, and combination treatment groups exhibited more severe clinical profiles and had fewer clear crystal days and more headache days than the no-treatment and oral medication-only groups. These results suggest that patients with more severe symptoms are more likely to use preventive treatment other than oral medications.

**Table 1:** Comparative analysis of baseline variables in MOH patients by classes and use of overused medication and preventive treatment types.

	Classes of overused medications						Use of overused medications				Types of preventive treatment					
groups	T-MOH (T, n = 66)	E-MOH (E, n = 29)	N-MOH (N, n = 74)	C-MOH (C, n = 57)	M-MOH (M, n = 79)	*p* value	Discontinuation (D, n = 143)	Reduction (R, n = 133)	Maintenance (M, n = 33)	*p* value	OBT-A (OB, n = 59)	Anti-CGRP mAb (CG, n = 53)	Combination of OBT-A and anti-CGRP mAb (BC, n = 27)	Oral medication only (OR, n = 149)	No preventive treatment (NO, n = 21)	*p* value
Age	43.0 (33.5–52.5)	51.0 (39.5–61.0)	39.5 (34.5–52.0)	48.0 (36.5–62.5)	48.0 (38.0–58.0)	**0.020** **T < C(0.046)** **T < M(0.024)** **N < C(0.025)** **N < M(0.010)**	45.0 (35.0–57.0)	44.0 (35.0–54.0)	49.0 (38.0–61.5)	0.125	44.0 (35.0–56.0)	42.0 (35.0–55.5)	51.0 (39.0–59.0)	45.0 (35.0–55.5)	40.0 (35.5–60.5)	0.722
Sex, female (%)	54 (81.8)	28 (96.6)	62 (83.8)	46 (80.7)	71 (89.9)	0.195	123 (86.0)	109 (82.0)	31 (93.9)	0.205	52 (88.1)	43 (81.1)	22 (81.5)	129 (86.6)	17 (81.0)	0.753
CCD	8.0 (0.0–10.0)	0.0 (0.0–6.5)	0.0 (0.0–7.3)	4.0 (0.0–10.0)	0.0 (0.0–10.0)	**0.002** **T > E(0.003)** **T > N(< 0.001)** **T > M(0.002)**	0.0 (0.0–5.0)	6.0 (0.0–10.0)	0.0 (0.0–5.0)	** < 0.001** **D < R(< 0.001)** **M < R(0.032)**	2.0 (0.0–7.0)	0.0 (0.0–10.0)	0.0 (0.0–6.0)	0.0 (0.0–10.0)	10.0 (1.5–15.0)	**0.042** **OB < NO(0.005)** **CG < NO(0.019)** **BC < NO(0.007)** **OR < NO(0.003)**
HD	23.5 (20.0–30.0)	30.0 (22.5–30.0)	30.0 (25.0–30.0)	28.0 (20.0–30.0)	30.0 (21.0–30.0)	**0.003** **T < E(0.029)** **T < N(< 0.001)** **T < M(0.004)**	30.0 (25.0–30.0)	24.0 (20.0–30.0)	30.0 (28.0–30.0)	** < 0.001** **D > R(< 0.001)** **M > R(0.001)**	29.0 (23.0–30.0)	30.0 (20.0–30.0)	30.0 (24.0–30.0)	30.0 (20.0–30.0)	20.0 (15.0–28.5)	**0.024** **OB > NO(0.001)** **CG > NO(0.011)** **BC > NO(0.007)** **OR > NO(0.002)**
SHD	8.0 (4.0–12.0)	6.0 (2.5–13.5)	10.0 (6.0–15.3)	10.0 (5.5–15.0)	10.0 (5.0–15.0)	**0.046** **T < N(0.015)** **E < N(0.014)**	10.0 (4.0–15.0)	10.0 (5.0–15.0)	10.0 (5.5–15.0)	0.208	10.0 (6.0–15.0)	8.0 (4.0–15.0)	8.0 (5.0–12.0)	10.0 (5.0–14.0)	8.0 (4.0–12.0)	0.224
DAM	15.0 (11.0–25.0)	25.0 (15.0–30.0)	20.0 (15.0–30.0)	20.0 (15.0– 30.0)	25.0 (15.0– 30.0)	**0.001** **T < E(0.004)** **T < N(0.002)** **T < C(0.031)** **T < M(< 0.001)**	**-**	**-**	**-**	**-**	20.0 (15.0–30.0)	20.0 (15.0–30.0)	28.0 (20.0–30.0)	20.0 (15.0–30.0)	20.0 (15.0–25.5)	**0.012** **OB < BC(0.002)** **BC > OR(0.001)** **BC > NO(0.002)**
PV	1.0 (0.0–1.0)	1.0 (1.0–1.0)	1.0 (0.0–2.3)	1.0 (0.0–2.0)	1.0 (0.0–1.0)	**0.004** **T < E(0.022)** **T < N(0.002)** **T < C(0.001)** **C > M(0.036)**	1.0 (0.0–2.0)	1.0 (0.0–1.0)	1.0 (0.0–1.0)	**0.022** **D > R(0.041)** **D > M(0.021)**	1.0 (0.0–2.0)	1.0 (0.0–2.0)	1.0 (1.0–1.0)	1.0 (0.0–2.0)	1.0 (0.3–2.5)	0.426
HV	1.0 (0.5–1.0)	1.0 (1.0–1.0)	1.0 (0.0–1.0)	1.0 (0.0–1.0)	1.0 (0.0–1.0)	0.182	1.0 (0.0–1.0)	1.0 (0.5–1.0)	1.0 (0.0–1.0)	0.102	0.5 (0.5–1.0)	1.0 (0.8–2.0)	1.0 (1.0–1.0)	1.0 (0.0–1.0)	1.0 (0.0–1.0)	**0.017** **CG > OR(0.017)** **CG > NO(0.034)** **BC > OR(0.034)** **BC > NO(0.031)**
MD	0.0 (0.0–0.0)	0.0 (0.0–0.5)	0.0 (0.0–0.0)	0.0 (0.0–0.0)	0.0 (0.0–0.0)	0.984	0.0 (0.0–0.0)	0.0 (0.0–0.0)	0.0 (0.0–0.0)	0.517	0.0 (0.0–1.0)	0.0 (0.0–0.0)	0.0 (0.0–1.0)	0.0 (0.0–0.0)	0.0 (0.0–0.0)	**0.008** **OB > OR(0.001)** **OB > NO(0.023)** **BC > OR(0.047)**
HIT-6	66.0 (61.8–72.0)	65.5 (63.3–69.8)	66.0 (62.0–72.0)	65.0 (60.5–70.0)	66.0 (64.0–72.0)	0.610	67.0 (62.8–72.0)	66.0 (62.5–70.0)	66.0 (61.0–71.5)	0.757	67.0 (64.0–72.0)	66.0 (63.0–70.0)	68.0 (64.0–76.0)	66.0 (62.0–70.0)	65.0 (60.0–70.5)	0.130
MIDAS	41.5 (13.8–86.3)	40.0 (23.3–73.8)	42.0 (20.0–83.3)	49.0 (20.5–108.5)	40.0 (16.0–100.0)	0.866	49.5 (22.8–90.5)	40.0 (14.5–90.0)	35.0 (10.0–107.0)	0.314	59.0 (30.0–138.8)	54.0 (25.5–110.0)	55.0 (22.0–100.0)	35.0 (16.5–80.0)	30.0 (8.0–56.0)	**0.012** **OB > OR(0.005)** **OB > NO(0.016)** **CG > OR(0.047)** **CG > NO(0.031)**
PHQ-9	10.0 (5.0–14.3)	8.0 (4.3–10.8)	10.0 (7.0–15.0)	9.0 (5.0–16.0)	12.0 (6.0–17.0)	0.143	10.0 (6.0–15.0)	10.0 (6.0–15.0)	10.0 (4.5–19.5)	0.977	12.5 (7.8–18.0)	11.0 (6.0–15.0)	11.0 (8.0–16.0)	9.0 (5.0–14.5)	8.0 (5.5–10.0)	**0.012** **OB > OR(0.009)** **OB > NO(0.004)** **CG > NO(0.038)** **BC > NO(0.016)**
GAD-7	7.0 (2.0–11.0)	3.5 (2.3–8.8)	6.0 (2.8–11.0)	6.0 (2.0–15.0)	5.0 (2.0–13.0)	0.529	6.5 (2.0–11.3)	6.0 (2.5–11.0)	7.0 (1.0–12.0)	0.764	7.0 (3.0–14.0)	5.0 (2.5–11.0)	7.0 (3.0–12.0)	6.0 (2.0–12.0)	3.0 (1.5–6.5)	0.110
MSQ	187.1 (123.1–242.3)	186.4 (147.4–237.9)	198.2 (140.1–227.7)	185.0 (110.8–234.5)	165.0 (110.9–208.3)	0.185	181.9 (116.1–220.6)	184.8 (140.2–230.1)	170.5 (112.1–230.8)	0.543	162.5 (113.6–195.8)	155.0 (90.6–204.9)	173.3 (108.6–221.9)	196.9 (140.7–238.6)	225.7 (188.5–249.5)	** < 0.001** **OB < OR(0.001)** **OB < NO(< 0.001)** **CG < OR(0.001)** **CG < NO(< 0.001)** **BC < NO(0.017)**

Kruskal–Wallis test and Mann–Whitney U test as post hoc test, data are presented as n (%) and a median (25–75th percentiles), and data from four patients with opioid-overuse headaches are not presented in the table.

Anti-CGRP mAb: anti-Calcitonin Gene-Related Peptide monoclonal antibody; CCD: crystal-clear days; C-MOH: combination analgesic-overuse MOH; DAM: days with acute medications per month; E-MOH: ergotamine-overuse MOH; GAD-7: general anxiety disorder-7; HD: headache days; HIT-6: headache impact test-6; HV: hospital visits per month; MD: Missed days; MIDAS: migraine disability assessment; M-MOH: MOH attributed to multiple drug classes; MOH: medication-overuse headache; MSQ: migraine-specific quality of life questionnaire; N-MOH: non-steroidal anti-inflammatory drug-overuse MOH; PHQ-9: patients health questionaire-9; OBT-A: OnabotulinumtoxinA; PV: Pharmacy visits per month; RD: reduction per month; SHD: severe headache days per month; T-MOH: triptan-overuse MOH. Significant values are in bold.

### Baseline vs. 3-month follow-up variables by classes of overused medications

M-MOH was the most common form of MOH, accounting for 25.6% (79/309) of all patients. Among MOH groups that overused a single medication, N-MOH was the most common, at 23.9% (74/309), whereas O-MOH was the rarest, at 1.3% (4/309) (Table 2). A GEE analysis, incorporating covariates including age, headache duration, CDH duration, and baseline monthly metrics such as crystal-clear days, headache days, and severe headache days, was conducted to assess the impact of classes of overused medications and the timing of visits (baseline vs. 3-month) on the clinical features of participants with MOH. Significant improvements were observed in all clinical parameters at the 3-month follow-up compared with those at baseline (Table 2). At the 3-month mark, the GEE model showed that patients in the M-MOH group had notably higher pharmacy visit days and HIT-6 and MIDAS scores than those in the T-MOH (*p* = 0.009), C-MOH (*p* = 0.003), and N-MOH (*p* = 0.04) groups, respectively. Additionally, the N-MOH group exhibited significantly lower MIDAS scores than the T-MOH (*p* = 0.025) and C-MOH (*p* = 0.001) groups. Except for the crystal-clear days per month (*p* = 0.018) and MIDAS scores (*p* = 0.018), no significant interaction was observed between the classes of overused medication and visits (Table 2). In essence, this suggests that the change in the crystal-clear days per month and MIDAS scores over time may differ depending on the type of medication.

**Table 2:** Evaluation of baseline and 3-month follow-up parameters in MOH patients based on classes of overused medications.

Variables	Baseline					3-month follow-up					*p* value*	*p* value†	*p* value‡
	T-MOH (n = 66)	E-MOH (n = 29)	N-MOH (n = 74)	C-MOH (n = 57)	M-MOH (n = 79)	T-MOH (n = 66)	E-MOH (n = 29)	N-MOH (n = 74)	C-MOH (n = 57)	M-MOH (n = 79)			
Crystal-clear days per month	8.0 (0.0–10.0)	0.0 (0.0–6.5)	0.0 (0.0–7.3)	4.0 (0.0–10.0)	0.0 (0.0–10.0)	18.0 (10.0–24.0)	17.0 (5.5–21.8)	18.0 (6.5–23.0)	16.5 (2.8–20.5)	10.0 (0.0–21.0)	** < 0.001**	0.583 M < E(0.032) T < E(0.022)	**0.018**
Headache days per month	23.5 (20.0–30.0)	30.0 (22.5–30.0)	30.0 (25.0–30.0)	28.0 (20.0–30.0)	30.0 (21.0–30.0)	11.0 (6.0–20.0)	13.0 (8.3–24.5)	12.0 (7.0–21.5)	10.0 (7.0–27.0)	20.0 (10.0–30.0)	** < 0.001**	0.300	0.092
Severe headache days per month	8.0 (4.0–12.0)	6.0 (2.5–13.5)	10.0 (6.0–15.3)	10.0 (5.5–15.0)	10.0 (5.0–15.0)	5.0 (2.0–10.0)	4.0 (0.0–8.5)	3.0 (0.0–8.0)	2.0 (0.0–6.3)	7.0 (2.0–10.0)	** < 0.001**	0.768	0.314
Acute medication days per month	15.0 (11.0–25.0)	25.0 (15.0–30.0)	20.0 (15.0–30.0)	20.0 (15.0– 30.0)	25.0 (15.0– 30.0)	7.0 (4.0–10.0)	7.0 (2.5–15.0)	7.0 (3.0–12.0)	6.0 (4.0–10.0)	10.0 (5.0–25.0)	** < 0.001**	0.215	0.084
Pharmacy visits per month	1.0 (0.0–1.0)	1.0 (1.0–1.0)	1.0 (0.0–2.3)	1.0 (0.0–2.0)	1.0 (0.0–1.0)	0.0 (0.0–0.0)	0.0 (0.0–0.0)	0.0 (0.0–0.0)	0.0 (0.0–0.0)	0.0 (0.0–0.0)	** < 0.001**	**0.009** **T < M(0.020)**	0.305
Hospital visits per month	1.0 (0.5–1.0)	1.0 (1.0–1.0)	1.0 (0.0–1.0)	1.0 (0.0–1.0)	1.0 (0.0–1.0)	0.0 (0.0–1.0)	0.0 (0.0–1.0)	0.0 (0.0–1.0)	0.0 (0.0–0.0)	0.0 (0.0–1.0)	** < 0.001**	0.079	0.088
Missed days per month	0.0 (0.0–0.0)	0.0 (0.0–0.5)	0.0 (0.0–0.0)	0.0 (0.0–0.0)	0.0 (0.0–0.0)	0.0 (0.0–0.0)	0.0 (0.0–0.0)	0.0 (0.0–0.0)	0.0 (0.0–0.0)	0.0 (0.0–0.0)	** < 0.001**	0.623	0.683
HIT-6	66.0 (61.8–72.0)	65.5 (63.3–69.8)	66.0 (62.0–72.0)	65.0 (60.5–70.0)	66.0 (64.0–72.0)	54.0 (51.0–61.0)	55.5 (48.0–62.8)	54.5 (48.5–62.8)	52.0 (45.8–58.3)	61.0 (52.0–66.0)	** < 0.001**	**0.041** **C < M(0.003)**	0.109
MIDAS	41.5 (13.8–86.3)	40.0 (23.3–73.8)	42.0 (20.0–83.3)	49.0 (20.5–108.5)	40.0 (16.0–100.0)	8.0 (0.0–31.0)	22.0 (4.3–33.8)	14.0 (2.3–39.3)	3.5 (0.0–21.0)	20.0 (5.0–41.0)	** < 0.001**	**0.011** **T < N(0.025)** **C < N(0.001)** **N < M(0.040)**	**0.018**
PHQ-9	10.0 (5.0–14.3)	8.0 (4.3–10.8)	10.0 (7.0–15.0)	9.0 (5.0–16.0)	12.0 (6.0–17.0)	6.0 (4.0–10.0)	5.0 (4.0–7.8)	7.0 (3.3–10.8)	4.0 (1.0–12.0)	9.0 (4.0–14.0)	** < 0.001**	0.304	0.549
GAD-7	7.0 (2.0–11.0)	3.5 (2.3–8.8)	6.0 (2.8–11.0)	6.0 (2.0–15.0)	5.0 (2.0–13.0)	4.0 (2.0–7.0)	2.0 (0.3–6.0)	4.0 (2.0–7.0)	2.0 (0.0–6.3)	5.0 (1.0–9.0)	** < 0.001**	0.224	0.863
MSQ	187.1 (123.1–242.3)	186.4 (147.4–237.9)	198.2 (140.1–227.7)	185.0 (110.8–234.5)	165.0 (110.9–208.3)	239.0 (198.2–284.2)	238.4 (213.6–283.1)	240.0 (210.0–282.9)	261.3 (230.6–287.6)	217.9 (141.2–265.0)	** < 0.001**	0.556	0.317

Data are presented as a median (25–75th percentiles). Data from four patients with opioid-overuse headaches are not presented in the table.

*p* value from generalized estimating equation method between groups, with age, headache duration, CDH duration, CDH to MOH period, and baseline of CCD, MHD, SHD, AMD, monthly pharmacy visits and the initial values of each scale before treatment as a covariate.

*p value for visit main effect.

^†^*p* value for class of medication main effect.

^‡^ *p* value for visit x medication (interaction) effect.

CDH: chronic daily headache; C-MOH: combination analgesic-overuse MOH; E-MOH: ergotamine-overuse MOH; GAD-7: general anxiety disorder-7; HIT-6: headache impact test-6; MIDAS: migraine disability assessment; M-MOH: MOH attributed to multiple drug classes; MOH: medication-overuse headache; MSQ: migraine-specific quality of life questionnaire; N-MOH: non-steroidal anti-inflammatory drug-overuse MOH; PHQ-9: patients health questionaire-9; T-MOH: triptan-overuse MOH. Significant values are in bold.

### Baseline vs. 3-month follow-up variables by use of overused medications

Over the 3-month period, 143 patients (46.3%) discontinued acute medications (discontinued group), 133 (43.0%) reduced their intake (median reduction: − 8.0, range, − 14.0 to − 3.0; reduction group), and 33 (10.7%) maintained their acute medication (maintenance group). GEE analysis revealed that the timing of visits (baseline vs. 3-month) significantly impacted on clinical parameters (Table 3). At the 3-month follow-up, patients in the discontinuation group reported fewer severe headache days than those in the maintenance group (*p* = 0.008). Regarding the impact of headache, the discontinuation group had lower HIT-6 scores than the maintenance (*p* = 0.002) and reduction (*p* = 0.001) groups. Furthermore, the discontinuation (*p* = 0.001) and reduction (*p* = 0.019) groups had higher MSQ scores than the maintenance group (Table 3).

**Table 3:** Evaluation of baseline and 3-month follow-up parameters in MOH patients based on use of overused medications.

Variables	Baseline			3-month follow-up			*p* value*	*p* value†	*p* value‡
	Discontinuation (D) (n = 143)	Reduction (R) (n = 133)	Maintenance (M) (n = 33)	Discontinuation (D) (n = 143)	Reduction (R) (n = 133)	Maintenance (M) (n = 33)			
Crystal-clear days per month	0.0 (0.0–5.0)	6.0 (0.0–10.0)	0.0 (0.0–5.0)	16.5 (7.8–23.0)	16.0 (7.0–22.0)	7.0 (0.0–20.0)	** < 0.001**	0.427 D > M(0.014) D > R(< 0.001)	** < 0.001**
Headache days per month	30.0 (25.0–30.0)	24.0 (20.0–30.0)	30.0 (28.0–30.0)	15.0 (7.8–23.0)	12.0 (7.0–20.0)	23.0 (8.0–30.0)	** < 0.001**	0.096	0.064
Severe headache days per month	10.0 (4.0–15.0)	10.0 (5.0–15.0)	10.0 (5.5–15.0)	3.0 (0.0–7.0)	5.0 (2.0–10.0)	5.0 (2.0–15.0)	** < 0.001**	**0.012** **D < M(0.008)**	0.051
Pharmacy visits per month	1.0 (0.0–2.0)	1.0 (0.0–1.0)	1.0 (0.0–1.0)	0.0 (0.0–0.0)	0.0 (0.0–0.0)	0.0 (0.0–0.0)	** < 0.001**	0.166	**0.030**
Hospital visits per month	1.0 (0.0–1.0)	1.0 (0.5–1.0)	1.0 (0.0–1.0)	0.0 (0.0–0.0)	0.0 (0.0–1.0)	0.0 (0.0–1.0)	** < 0.001**	0.354	0.201
Missed days per month	0.0 (0.0–0.0)	0.0 (0.0–0.0)	0.0 (0.0–0.0)	0.0 (0.0–0.0)	0.0 (0.0–0.0)	0.0 (0.0–0.0)	** < 0.001**	0.750	0.704
HIT-6	67.0 (62.8–72.0)	66.0 (62.5–70.0)	66.0 (61.0–71.5)	52.0 (46.0–60.0)	57.5 (52.0–64.0)	60.5 (53.0–71.3)	** < 0.001**	**0.001** **D < M(0.002)** **D < R(0.001)**	** < 0.001**
MIDAS	49.5 (22.8–90.5)	40.0 (14.5–90.0)	35.0 (10.0–107.0)	10.5 (0.0–26.3)	17.5 (0.8–40.0)	20.0 (5.8–60.0)	** < 0.001**	0.839	0.356
PHQ-9	10.0 (6.0–15.0)	10.0 (6.0–15.0)	10.0 (4.5–19.5)	6.0 (2.5–10.5)	6.5 (3.0–11.0)	9.5 (6.0–20.0)	** < 0.001**	0.275 D < M(0.030)	**0.024**
GAD-7	6.5 (2.0–11.3)	6.0 (2.5–11.0)	7.0 (1.0–12.0)	2.5 (1.0–7.0)	4.0 (1.0–7.0)	6.0 (1.5–17.0)	** < 0.001**	0.598	0.598
MSQ	181.9 (116.1–220.6)	184.8 (140.2–230.1)	170.5 (112.1–230.8)	252.5 (213.6–286.2)	239.2 (197.6–273.2)	197.1 (119.3–249.8)	** < 0.001**	**0.007** **D > M(0.001)** **R > M(0.019)**	**0.018**

Data are presented as a median (25–75th percentiles).

*p* value from generalized estimating equation method between groups, with headache onset age, headache duration, CDH duration, MOH duration, CDH to MOH period, and baseline of CCD, MHD, monthly pharmacy visits and the initial values of each scale before treatment as a covariate.

*p value for visit main effect.

^†^*p* value for management strategy main effect.

^‡^ *p* value for visit x treatment strategy (interaction) effect.

GAD-7: general anxiety disorder-7; HIT-6: headache impact test-6; MIDAS: migraine disability assessment; MSQ: migraine-specific quality of life questionnaire; PHQ-9: patients health questionaire-9. Significant values are in bold.

Regarding the interaction between visits and the use of overused medications, GEE analysis showed significant interactions for the following parameters: change in crystal-clear days per month, monthly pharmacy visits, and HIT-6, PHQ-9, and MSQ scores. This implies that the clinical outcomes at the 3-month follow-up were influenced by the use of overused medications in relation to the timing of the visit (Table 3).

### Baseline vs. 3-month follow-up variables by types of preventive treatments

During the 3-month follow-up period of the 309 participants, 21 declined preventive treatment, leaving 288 participants who received preventive interventions (Table 4). None of the participants switched their initial treatment plan during the entire 3-month study period. The oral medication-only group was the most common, encompassing 149 (51.7%) participants, followed by the OBT-A group with 59 (20.5%) participants and anti-CGRP mAb group with 53 (18.4%) participants. A smaller cohort of 27 participants (9.4%) received a combination of the OBT-A and anti-CGRP mAb. All participants in the OBT-A and anti-CGRP mAb groups were administered oral preventive medications.

**Table 4:** Evaluation of baseline and 3-month follow-up parameters in MOH patients based on types of preventive treatments.

Variables	Baseline					3-month follow-up					*p* value*	*p* value†	*p* value‡
	OBT-A (OB) (n = 59)	Anti-CGRP mAb (CG) (n = 53)	OBT-A + anti-CGRP mAb (BC) (n = 27)	Oral med only (OR) (n = 149)	No preventive treatment (NO) (n = 21)	OBT-A(OB) (n = 59)	Anti-CGRP mAb (CG) (n = 53)	OBT-A + anti-CGRP mAb (BC) (n = 27)	Oral med only (OR) (n = 149)	No preventive treatment (NO) (n = 21)			
Crystal-clear days per month	2.0 (0.0–7.0)	0.0 (0.0–10.0)	0.0 (0.0–6.0)	0.0 (0.0–10.0)	10.0 (1.5–15.0)	10.0 (3.0–20.5)	20.0 (7.0–25.0)	10.0 (0.0–24.3)	15.0 (7.0–21.0)	20.0 (17.0–25.5)	** < 0.001**	0.249	0.518
Headache days per month	29.0 (23.0–30.0)	30.0 (20.0–30.0)	30.0 (24.0–30.0)	30.0 (20.0–30.0)	20.0 (15.0–28.5)	20.0 (10.5–27.0)	10.0 (5.0–20.0)	20.0 (5.8–30.0)	15.0 (8.0–20.0)	10.0 (4.5–10.0)	** < 0.001**	**0.007** **OR > NO(0.033)** **BC > NO(0.016)** **OB > NO(0.003)** **OB > CG(0.009)**	**0.010**
Severe headache days per month	10.0 (6.0–15.0)	8.0 (4.0–15.0)	8.0 (5.0–12.0)	10.0 (5.0–14.0)	8.0 (4.0–12.0)	7.0 (4.5–14.5)	2.0 (0.0–5.0)	5.5 (2.8–10.0)	4.0 (1.0–9.5)	4.0 (2.0–6.5)	** < 0.001**	**0.001** **OB > OR(0.014)** **BC > OR(0.039)** **OB > CG(0.007)** **BC > CG(0.011)**	**0.026**
Acute medication days per month	20.0 (15.0–30.0)	20.0 (15.0–30.0)	28.0 (20.0–30.0)	20.0 (15.0–30.0)	20.0 (15.0–25.5)	10.0 (4.0–16.0)	5.0 (3.0–10.0)	12.0 (4.5–30.0)	7.0 (4.0–12.0)	6.0 (3.0–9.0)	** < 0.001**	0.078	0.099
Pharmacy visits per month	1.0 (0.0–2.0)	1.0 (0.0–2.0)	1.0 (1.0–1.0)	1.0 (0.0–2.0)	1.0 (0.3–2.5)	0.0 (0.0–0.0)	0.0 (0.0–0.0)	0.5 (0.0–1.0)	0.0 (0.0–0.0)	0.0 (0.0–0.5)	** < 0.001**	0.309	0.415
Hospital visits per month	0.5 (0.5–1.0)	1.0 (0.8–2.0)	1.0 (1.0–1.0)	1.0 (0.0–1.0)	1.0 (0.0–1.0)	0.0 (0.0–1.0)	0.0 (0.0–0.0)	1.0 (0.0–1.0)	0.0 (0.0–0.0)	1.0 (0.0–1.0)	** < 0.001**	0.207 NO > OR(0.044) NO > CG(0.037)	**0.028**
Missed days per month	0.0 (0.0–1.0)	0.0 (0.0–0.0)	0.0 (0.0–1.0)	0.0 (0.0–0.0)	0.0 (0.0–0.0)	0.0 (0.0–0.0)	0.0 (0.0–0.0)	0.0 (0.0–0.0)	0.0 (0.0–0.0)	0.0 (0.0–0.0)	** < 0.001**	0.094 NO > OR(0.044) NO > CG(0.037)	**0.005**
HIT-6	67.0 (64.0–72.0)	66.0 (63.0–70.0)	68.0 (64.0–76.0)	66.0 (62.0–70.0)	65.0 (60.0–70.5)	60.5 (52.5–67.5)	55.0 (49.5–60.5)	63.0 (48.0–66.5)	53.0 (50.0–60.0)	61.0 (54.0–69.3)	** < 0.001**	**0.015** **OB > OR(0.049)** **OB > CG(0.002)**	0.089
MIDAS	59.0 (30.0–138.8)	54.0 (25.5–110.0)	55.0 (22.0–100.0)	35.0 (16.5–80.0)	30.0 (8.0–56.0)	23.0 (3.5–59.8)	5.0 (0.0–30.5)	14.0 (0.0–57.5)	10.5 (2.5–25.0)	27.0 (12.0–61.0)	** < 0.001**	0.186	0.245
PHQ-9	12.5 (7.8–18.0)	11.0 (6.0–15.0)	11.0 (8.0–16.0)	9.0 (5.0–14.5)	8.0 (5.5–10.0)	8.0 (4.0–13.8)	6.0 (3.3–9.0)	7.0 (3.0–10.0)	7.0 (3.0–11.3)	5.5 (4.0–12.8)	** < 0.001**	0.249	0.107
GAD-7	7.0 (3.0–14.0)	5.0 (2.5–11.0)	7.0 (3.0–12.0)	6.0 (2.0–12.0)	3.0 (1.5–6.5)	6.0 (2.0–9.5)	3.0 (1.5–6.5)	2.0 (0.0–6.0)	3.5 (1.0–7.0)	1.5 (0.0–7.0)	** < 0.001**	0.535	0.128
MSQ	162.5 (113.6–195.8)	155.0 (90.6–204.9)	173.3 (108.6–221.9)	196.9 (140.7–238.6)	225.7 (188.5–249.5)	220.1 (166.2–257.1)	253.0 (213.2–282.8)	224.8 (125.7–287.1)	252.1 (213.0–284.8)	220.8 (144.1–259.1)	** < 0.001**	**0.036** **CG > OR(0.004)** **CG > NO(< 0.001)** **CG > OB(0.013)**	**0.001**

Data are presented as a median (25–75th percentiles).

*p* value from generalized estimating equation method between groups, with CDH to MOH period and baseline of AMD, MIDAS, PHQ-9, MSQ and the initial values of each scale before treatment as a covariate.

*p value for visit main effect.

^†^*p* value for preventive therapy main effect.

^‡^ *p* value for visit x preventive medication (interaction) effect.

Anti-CGRP mAb: anti-Calcitonin Gene-Related Peptide monoclonal antibody; GAD-7: general anxiety disorder-7; HIT-6: headache impact test-6; MIDAS: migraine disability assessment; MSQ: migraine-specific quality of life questionnaire; PHQ-9: patients health questionaire-9; OBT-A: OnabotulinumtoxinA. Significant values are in bold.

When considering the types of preventive treatments based on the groups of acute medication usage, among the 143 individuals in the discontinued group, almost all patients (141 patients, 98.6%) agreed to undergo preventive treatment. Specifically, 83 patients (58%) received only oral medications, 27 patients (18.9%) received OBT-A, 20 patients (14%) received anti-CGRP mAb, and 12 patients (8.4%) received both OBT-A and anti-CGRP mAb. In the reduction group, aside from 19 individuals, most agreed to undergo preventive treatment (114 patients, 85.7%). Among them, 47 patients (35.3%) belonged to the oral medication-only group, followed by 28 patients (21.1%) in the OBT-A group, 26 patients (19.5%) in the anti-CGRP mAb group, and 13 patients (9.8%) receiving both treatments. In the maintenance group, the majority opted for oral medication-only treatment (19 patients, 57.6%). However, anti-CGRP mAb treatment was the second most common choice, with 7 individuals (21.2%), followed by 4 individuals (12.1%) receiving OBT-A, and 2 individuals (6.1%) receiving both.

After 3-month of treatment, the no-treatment group showed significantly fewer headache days per month than the other treatment groups. The oral medication-only group experienced fewer severe headache days per month and had lower HIT-6 scores than the OBT-A group. Notably, the anti-CGRP mAb group had a higher MSQ score than the other treatment groups (Table 4). Subsequent GEE analysis showed a significant interaction between the types of preventive treatments and changes in clinical characteristics such as headache days per month, severe headache days per month, hospital visits per month, missed days per month, and MSQ scores, suggesting that the variability in these outcomes over time may be influenced by the type of preventive treatments (Table 4).

## Discussion

The main findings of this 3-month prospective observational study are as follows: (1) A significant improvement in MOH was observed at the 3-month follow-up from baseline. This improvement was consistent regardless of the classes and use of overused medications and types of preventive treatments. (2) Some clinical characteristics at 3 months including headache and severe headache days per month, pharmacy visits per month, as well as HIT-6, MIDAS, and MSQ scores, exhibited significant differences according to the classes and use of overused medications and types of preventive treatments. (3) The classes and use of overused medications and types of preventive treatment showed significant interactions with some clinical characteristics of MOH at the 3-month mark. Specifically, patients overusing multiple drug classes experienced a higher impact of headaches than those overusing combined analgesics at baseline and 3 months. Furthermore, some clinical features of patients who maintained their use of overused acute medications were more severe than those of patients who either discontinued or reduced their use of overused acute medications after the 3-month period.

MOH is a serious condition that imposes approximately 3 times the burden on individuals as migraine and approximately 10 times the burden of tension-type headaches^10,25^. Given that adequate MOH treatment provides significant benefits to individuals and society, healthcare systems should prioritize the treatment of MOH^26^. The present study validated previous reports that MOH treatment significantly improved patients’ disability and quality of life and clinical characteristics of headaches, regardless of the classes of overused medications^27,28^. Our results further support the urgent need for the diagnosis and treatment of MOH from the patient’s perspective.

Notably, our results showed that patients with M-MOH had more profound disabilities and a higher impact of headache than patients who overused single-class medications at the 3-month follow-up. In our previous study, the M-MOH group displayed more severe clinical characteristics than the single-class MOH groups^14^. Considering these, multidrug overuse negatively influences not only MOH clinical presentations at baseline but also patient prognoses; however, the underlying mechanisms of this difference remain elusive^29^. Nevertheless, the ongoing interplay of multiple drug classes might impact multiple pathways, reducing the threshold for headache expression and leading to the development of more severe forms of headache. Our previous research indicated a faster transition from CDH onset to MOH in the M-MOH group compared to single-class MOH groups^14^. This accelerated progression implies that the M-MOH not only exacerbates symptoms but is also linked to a higher risk of headache chronification and shortens the time needed for MOH development.

Despite the high prevalence of MOH, a universally accepted treatment strategy has yet to be established^1,8^. Attempts to create an internationally recognized evidence-based guideline are ongoing; however, inconsistencies persist in various aspects of MOH treatment, leading to significant differences in therapeutic approaches^30,31^. One of the major debates is the use of overused medications^8,32^. Withdrawal treatment can transform a chronic headache back into an episodic form in roughly 70% of patients^33,34^. A study by the Danish Headache Center demonstrated that a 2-month outpatient detoxification program, which did not permit the use of any acute migraine medication for breakthrough pain, was more successful than allowing limited use of acute migraine medication with a maximum of 2 days per week^35^. A study that compared three MOH treatment strategies—simultaneous withdrawal and preventive treatment, preventive treatment without withdrawal, and withdrawal with the possibility of delayed preventive treatment—demonstrated that all strategies were effective in managing MOH. However, the combination of withdrawal and preventive treatment was more effective than preventive treatment alone^35^. The present study highlighted that patients who maintained their use of overused acute medications had less favorable outcomes than those who discontinued or reduced their use of overused acute medications. This finding implies that stopping or reducing the use of overused medications could be more beneficial than maintaining medication use for the treatment of MOH.

In recent decades, significant advances have been made in preventive treatments for patients with MOH. In recent randomized controlled trials, OBT-A and monoclonal antibodies targeting CGRP or its receptor have been shown to be effective in the treatment of MOH^36,37^. The present study showed that patients with severe clinical profiles were more likely to opt for OBT-A and anti-CGRP mAbs, in addition to oral preventive treatment. Encouragingly, at the 3-month follow-up, patients who received OBT-A and anti-CGRP mAb treatments displayed considerable clinical improvement. Our study also showed that preventive treatment types had a significant interaction with changes in certain clinical outcomes at the 3-month mark, suggesting that treatment results may differ according to the type of preventive treatment chosen.

Results from the Medication Overuse Treatment Strategy (MOTS) trial demonstrates that clinically meaningful reductions in moderate to severe headache days with migraine preventive medication, which were not inferior to limiting symptomatic medication compared to continuation of the overused medication with no maximum limit ^38^. In contrast, current study reported a significant reduction in severe headache days per month in the discontinued group compared to the maintenance group (*p* = 0.008) at the 3-month follow-up (Table 3). This difference in results could be attributed to both differences in sample size and variations in how acute medication groups were defined. While the MOTS trial categorized patients into switching group (≤ 2 times per week) versus no limit group based on symptomatic medication use, this study stratified patients into discontinuation, reduction, and maintenance groups, reflecting differences in acute medication use. Furthermore, the results of the MOTS trial emphasize that migraine preventive medication has a more significant impact on reducing moderate to severe headache days compared to treatment involving adjustments to acute medication. In the trial, topiramate, OBT-A, and amitriptyline were randomly assigned as preventive medications, and there were no clear differences in clinical profiles between the three groups at baseline. However, in our study, patients were allowed to choose their preventive medication according to their preferences. In this observational study, we observed overall favorable headache-related profiles in the group not selecting preventive treatment at baseline. Thus, it is presumed that the selection of additional preventive medications beyond oral medication may have influenced worse headache-related profiles. However, it is worth noting that in the discontinued group, the proportion of patients receiving oral medication-only preventive treatment was relatively higher compared to the reduction group, suggesting that the treatment approach regarding acute medication use may also influence the choice of preventive treatment.

We examined the changes in headache-related variables in MOH patients over a 3-month follow-up period, considering the class of overused medications, the use of overused medications, and the type of preventive medications. The no-treatment group had fewer headache days, more crystal-clear days, and less disability due to headache (MIDAS) at baseline compared to the oral medication only, OBT-A, or anti-CGRP mAb groups (Table 1). At the 3-month follow-up, the no-treatment group still had fewer headache days than the other groups, but there was no difference in crystal-clear days and MIDAS between the no-treatment group and the groups that received preventive treatment (Table 3). These results suggest that the use of preventive medications, such as OBT-A and anti-CGRP mAbs, is more effective than not using preventive medications in treating headaches over a 3-month period. The GEE analysis of our study found that there was no significant interaction between crystal-clear days and MIDAS in the groups that received preventive treatment, including those who did not receive preventive treatment. This lack of significant interaction could be attributed to the subdivision of preventive treatment groups, which resulted in insufficient sample sizes for each group.

The present study had some limitations. First, although this was a prospective study, blinded or controlled trials were not incorporated for any particular treatment. This could affect the study's credibility, broader relevance, and capability to deduce direct cause-and-effect relationships. Second, a potential selection bias emerged as our focus remained on patients who concluded the 3-month evaluation. Older patients and individuals with long-standing MOH may face a higher likelihood of dropping out and having incomplete data episodes owing to the challenges associated with frequent assessments and ongoing outpatient visits^39,40^. Third, the data originated from a registry study with a set sample size, and certain subgroups may have been underrepresented, thereby weakening the analysis. This could explain the absence of significant results in certain subgroup analyses. Fourth, the follow-up period of the present study was relatively short. Many follow-up studies of MOH had a 6-month to 1-year follow-up period, whereas our study had a follow-up period of only 3 months. However, a study conducted in seven European and South American countries found significant improvements in monthly headache days and comorbid anxiety and depression after three months of initiating MOH treatment. Nonetheless, there was no significant change in symptoms were noted after that. In a randomized controlled trial comparing preventive treatment and withdrawal of overused medications of MOH, the number of monthly migraine days, days with acute medications and pain intensity improved significantly after 2-month treatment, with no further difference at 6-month. Therefore, even with a 3-month follow-up period, our study should be able to capture most of the changes after MOH treatment. Finally, the inclusion of patients solely from seven specialized referral hospitals in a designated area might skew the representation, possibly resulting in a selection bias towards patients with more severe headaches compared to those attending primary clinics.

In conclusion, this prospective registry study found a significant improvement in MOH after a 3-month treatment from the baseline. Nevertheless, some clinical characteristics of MOH significantly differed according to the classes and use of overused medications and types of preventive treatments at 3 months. GEE analyses revealed significant interactions between the classes and use of overused medications and types of preventive treatment and some clinical characteristics at the 3-month mark, suggesting that the clinical outcomes of MOH treatment may be influenced by overused medications, use of overused medications, and types of preventive treatment.

## Data Availability

Anonymized demographic and clinical data not published within this article will be made available by request from qualified investigators.
